# Cross-sectional and longitudinal associations between sleep, sedentary behaviour and physical activity with adiposity and cardio-respiratory fitness in school-aged children: a compositional data analysis

**DOI:** 10.1186/s44167-025-00082-y

**Published:** 2025-07-11

**Authors:** Aaron Miatke, Tim Olds, Carol Maher, Francois Fraysse, Dorothea Dumuid

**Affiliations:** 1 Alliance for Research in Exercise, Nutrition and Activity (ARENA), Allied Health and Human Performance, University of South Australia, Adelaide, 5000 Australia; 2 Centre for Adolescent Health, Murdoch Children’s Research Institute, Parkville, 3052 Australia

**Keywords:** Time use, Movement behaviours, Compositional data analysis, CoDA, Fitness, Adiposity, Obesity, Accelerometry, MVPA, Children

## Abstract

**Background:**

Time spent in sleep, sedentary behaviour (SB), light physical activity (LPA) and moderate-to-vigorous physical activity (MVPA) all impact child health. The aim of this study was to investigate cross-sectional and longitudinal associations between time use and adiposity and cardio-respiratory fitness (CRF) in school-aged children.

**Methods:**

Cross-sectional (*n* = 281) and longitudinal (*n* = 305) data were used from the Life on Holidays study with assessments over three time periods (two consecutive school years, and the interleaving summer holiday period). 24-h time use was measured using wrist-worn accelerometers. Adiposity was assessed as BMI z-score from measured weight and height, using World Health Organization reference data, and from body fat percentage (%BF) measured via bioelectrical impedance. CRF was operationalised as estimated VO_2_max from the 20-m shuttle run test. Compositional data analysis in conjunction with linear mixed-effects models was used to investigate the associations between time-use composition and outcomes after controlling for covariates. Cross-sectional models used baseline measures, and longitudinal models used rates of change in each outcome. Sensitivity analyses explored relationships for moderate physical activity (MPA) and vigorous physical activity (VPA) separately.

**Results:**

In cross-sectional analyses, time spent in MVPA was favourably associated with all adiposity and fitness outcomes (all *p* < 0.01) whereas time in LPA was unfavourably associated with all outcomes (all *p* < 0.01). Sleep was favourably associated with %BF, whereas SB was unfavourably associated (both *p* = 0.02). In longitudinal models, only MVPA was significantly associated with any of the three outcomes. 30 min/day more time spent in MVPA was associated with a decrease in %BF rate of change (-0.60 to -0.48) when time was reallocated from LPA or SB, and with an increase in VO_2_max (+ 0.89 to + 1.01) when time was reallocated from any other behaviour. Sensitivity analyses showed VPA was significantly associated with changes in %BF and VO_2_max.

**Conclusions:**

All behaviours displayed cross-sectional associations with adiposity and CRF. However, only MVPA (and in particular VPA) was significantly associated with changes in %BF or VO_2_max in longitudinal models. Future efforts should focus on increasing participation in MVPA with school-aged children to see the most benefit to health.

**Supplementary Information:**

The online version contains supplementary material available at 10.1186/s44167-025-00082-y.

## Background

Adiposity and cardio-respiratory fitness (CRF) are two key indicators of overall health and well-being in children [1, 2]with high adiposity and low fitness in childhood also linked to increased morbidity in adulthood [3]. However, despite various health promotion efforts aimed to reduce the prevalence of childhood obesity, it remains a global problem affecting over 340 million children worldwide [4]. Evidence also suggests that CRF is lower in children today than a generation ago [5]. Addressing these dual challenges of childhood obesity and declining fitness remains a critical public health priority.

The way children spend their time - the amount of time spent sleeping, being sedentary, or physically active - has long been associated with adiposity and fitness levels in children [6–8]. Historically, these behaviours have been studied independently, often neglecting their inherent compositional nature. Increasing time spent in one behaviour must come at the expense of time spent in another, highlighting the need for analytical approaches that account for this interdependence. Recently, studies have used compositional data analysis (CoDA) to explore the joint effects of these behaviours on health. A recent review of CoDA studies found that reallocating time towards moderate-to-vigorous physical activity (MVPA) generally showed the strongest favourable associations with health, with similar benefits seen regardless of which behaviours were substituted [9]. However, it was also noted that some smaller benefits may be seen when reallocating time among other behaviours, for example, when reallocating time from sedentary behaviour (SB) to sleep. These findings were supported by a recent meta-analysis of CoDA studies in children, which came to a similar conclusion [10]. MVPA showed the most consistent favourable associations for health, with SB and light physical activity (LPA) showing unfavourable associations for markers of adiposity. The relationship between time-use composition and fitness were less clear, with both reviews noting that few studies have studied this relationship making it difficult to draw firm conclusions. Another recent systematic review of CoDA studies investigating the association between time-use composition and adiposity in children also suggested that engaging in more MVPA and sleep was associated with lower adiposity, while spending more time in SB or light physical activity (LPA) is associated with higher adiposity [11]. However, the majority of studies included in this review used zBMI as an indicator for adiposity levels rather than a more direct measure of adiposity, such as percent body fat (%BF) or fat mass index.

One limitation of all three of the aforementioned reviews, and other non-CoDA reviews [12, 13] that have reported similar findings, is that the vast majority of studies included were cross-sectional in nature. This limitation is true for most studies that have used CoDA to investigate associations between time use and health to date.

Importantly, this limits the ability to draw conclusions about the relationship between time-use compositions and health, with no empirical changes in health observed in these studies. This leaves a critical gap in our understanding of how time-use composition influences health trajectories. Therefore, this study aims to (1) examine cross-sectional associations between time-use composition and CRF and adiposity in school children, and extend this by (2) investigating longitudinal associations between time-use composition with empirical changes in health outcomes (BMI, %BF and VO_2_max) across three distinct time periods. Based on previous literature we hypothesised that spending more time in MVPA and sleep would be favourably associated with all health outcomes, and time spent in SB and LPA would be unfavourably associated with all health outcomes.

## Methods

### Study design and participants

This study used cross-sectional and longitudinal data from the Life on Holidays (LoH) study [14]a 2-year longitudinal cohort study tracking changes in children’s time use, fitness and body composition across school year and summer holiday periods. The study sampled 24 primary schools in Adelaide, Australia, using a stratified random sampling approach. Schools were selected within socio-economic tertiles based on the Index of Community Socio-Educational Advantage, which considers parental education, occupation, and school location [15]. If a school agreed to participate, all Grade 4 children were invited to participate in the study. Parents provided written informed consent for their child to participate, and children gave verbal assent to participate. Recruitment continued in this manner until at least 100 children were recruited from each SES tertile. In total, 24 schools participated in the LoH study (21% uptake at the school level), with 381 child participants (43% uptake at the participant level). Between 259 and 305 participants (depending on outcome and analysis) had complete time use, outcome and covariate data and were included in the current study.

Data were collected over five timepoints, spanning two school years. In Australia, the school year starts in January, and the summer holidays (between two school years) occur from mid-December to late January. Timepoint 1 (first term of Grade 4) and 2 (last term of Grade 4) measures were collected at the beginning and end of the first school year, respectively; Timepoint 3 occurred in the summer holiday period following Grade 4; Timepoint 4 and 5 measures were collected at the beginning and end of the second school year (first and last term of Grade 5), respectively. Measurements for in-school timepoints (Timepoints 1, 2, 4 and 5) were typically within a month of the start/end of the school holiday period. Time use was measured at all 5 timepoints, outcome variables (BMI, %BF, VO_2_max) were measured during all in-school timepoints (timepoints 1,2,4 and 5). Covariates were measured at baseline only. The study ran from February 2019 to December 2022.

### Measures

#### Dependent variables

The dependent variables were adiposity (BMI z-score (zBMI) and percent body fat (%BF)) and CRF (VO_2_max). Standing height was measured using a Seca 213 stadiometer (Seca, Hamburg, Germany). Weight and %BF were measured using InBody 270 Bioelectrical Impedance Analyser scales (InBody, Seoul, South Korea), without shoes and in light clothing. Measures were taken twice, unless the two measures differed by > 0.5 cm, 0.5 kg or 1%, in which case a third measure was taken. A mean of the first two, or median of the three measures were used in subsequent analyses. BMI was calculated from measured height and weight with age- and sex-standardised zBMI scores derived using World Health Organization reference data [16]. The InBody provides a valid (*r* = 0.69–0.79) and reliable (CVintra = 3%) estimate of body fat when compared to gold standard underwater weighing in this age group [17]. CRF was assessed using the 20-m shuttle run test [18] (20mSRT). This is a widely used test that involves children continuously running between two lines, 20 m apart. Children follow audio cues in order to keep up with gradually increasing speeds until they can no longer maintain the pace required for two consecutive levels. The level attained in the 20mSRT was then used to estimate VO_2_max using the equation published by Nevill et al. (2021) [19]. The 20mSRT has good test-retest reliability [20] (*r* = 0.78–0.93) and criterion validity [21] (*r* = 0.78) compared to gold standard gas-analysis testing. All dependent variables were measured by trained research assistants.

In cross-sectional models, zBMI, %BF and estimated VO_2_max at Timepoint 1 were used as the dependent variables. In longitudinal models, annualised rates of change (RoC) across the three measurement periods (year 1, holidays, year 2) for each health outcome (zBMI, %BF and VO_2_max) were used as the dependent variables. Rates of change were calculated by first calculating the absolute change in outcome between timepoints, e.g., year 1 = (∆T1-T2); holidays = (∆T2-T4); year 2 = (∆T4-T5). Absolute changes were then divided by the number of days between measurements for each child and then multiplied by 365 to express as annualised rates of change. This was done to aid interpretation and allow comparability of changes across time periods with differing durations.

### Independent variables

#### Accelerometry

Time spent sleeping, sitting, in LPA and MVPA, collectively referred to as time-use composition, were the independent variables. Time use was measured via wrist-worn GENEActiv accelerometers (Activinsights, Cambridgeshire, UK) which were worn 24/h day for 7 consecutive days at each timepoint. The GENEActiv has good intra- and inter-instrument reliabilities [22] (CVintra = 1.4% and CVinter = 2.1%), good test–retest reliability [23] (ICC > = 0.7 with 3–5 days wear) and excellent convergent validity [24] (*r* = 0.98). Devices were set to record at 50 Hz. For in-school timepoints, trained research assistants distributed and collected the devices. For timepoint 3 during the school-holidays, accelerometers were distributed to children on the final week of the school year, with children instructed to start wearing the device on their first day of holidays.

Accelerometry data were processed in Matlab using the Cobra processing software used previously in other studies [25]. Data were first collapsed in 60-second epochs. Device removals were identified using a log and excluded from analysis. Extended periods of non-wear not reported in logs were identified visually during data processing and manually excluded. Sleep logs were used to identify time in bed and get-up time, and the algorithm proposed by van Hees et al. (2015) [26] was then used to compute sleep characteristics (for this study, Total Sleep Time was used). Subsequently, waking-wear time was classified as either SB, LPA or MVPA using previously published cutpoints by Philips et al. (2013) [27]. A day was considered valid if it contained at least 600 min of waking-wear time. Four valid days, including at least one weekend day, were needed for a participant’s accelerometry data to be considered valid at each timepoint. Average daily time spent in each behaviour were determined by weighting the weekdays and weekend days 5:2 to account for differences in the way children may spend their time during weekdays and weekend days [28–30]. In cross-sectional models, time use from Timepoint 1 was used as the independent variable. In longitudinal models, composite year 1, holiday and year 2 time-use compositions were constructed by averaging time use from Timepoint 1 and Timepoint 2 for year 1, and Timepoint 4 and Timepoint 5 for year 2. If a participant only had valid accelerometry data from a single timepoint within a year (e.g., only at Timepoint 1 and not at Timepoint 2), these values were used to represent time use over that period. Average time-use compositions for year 1 and year 2 were created to allow for a single time-use composition to be used the independent variable, aligning with a corresponding outcome-change interval as the dependent variable.

### Covariates

Covariates included sex, socio-economic status (SES) and baseline age (continuous, calculated from date of birth). Sex and age were obtained via a parental questionnaire conducted at baseline. SES was determined via the Index of Relative Socio-economic Disadvantage (IRSD). The IRSD is a composite socio-economic indicator calculated by the Australian Government that is reported at the postcode level and considers a variety of indicators related to the economic and social conditions of people residing in households within each area such as income, occupation and education [31]. IRSD deciles (1–10 with 1 representing the most disadvantaged) were grouped in low SES (deciles 1–2); Mid SES (deciles 3:7) and high SES (deciles 8–10). IRSD was chosen over other indicators of SES such as parental reported income or education levels in order to maximise sample size without the need for imputation.

### Compositional data analysis

To overcome the inherent co-dependency of times spent in different movement behaviours, CoDA was undertaken. Following published CoDA methods [32]the four-part composition consisting of daily time spent in sleep, SB, LPA and MVPA was expressed as 3 isometric log-ratio (*ilr*) coordinates. A specific type of *ilr* transformation known as pivot coordinates was undertaken using a sequential binary partition process, such that the first *ilr* coordinate represented the dominance of a particular behaviour relative to the geometric mean of the remaining behaviours (e.g., sleep vs. the geometric mean of SB + LPA + MVPA). Four unique sets of *ilr* coordinates were constructed by permuting the order of behaviours in the sign matrix such that each behaviour was iteratively represented as the numerator of the first *ilr* coordinate resulting in four equivalent sets of *ilr* coordinates. Sensitivity analyses were also conducted using a five-part composition where time in MVPA was separated into moderate physical activity (MPA) and vigorous physical activity (VPA) and therefore resulted in four *ilr* coordinates. Before constructing *ilr* coordinates, compositions were first checked for the presence of zeroes. 0.4% and 2.5% of observations had observed zero values for VPA in cross-sectional and longitudinal models, respectively. These were replaced using the log-ratio expectation-maximisation algorithm [33] before *ilr* coordinates were constructed.

### Statistical analyses

All statistical analyses were performed using R 4.3.1 (R Foundation for Statistical Computing, Vienna, Austria) using the *lme4* [34], *nlme* [35] and *compositions* [36] packages with statistical significance set at *p* < 0.05. Formal power analysis methods have not been developed specific to CoDA. However, post-hoc power analysis for 10 regressors, an alpha of 0.05, power of 0.80, and a small effect size (Cohen’s d = 0.1) indicated that at least 172 participants were required. Descriptive statistics for baseline participant characteristics were reported as means and standard deviations for continuous variables and frequency and proportion for categorical variables. Baseline demographic characteristics of those participants included in the analyses were compared to those not included via a two-sided t-test for continuous variables (age) and a chi-squared test for categorical variables (sex, SES). Multilevel mixed-effects models were used to investigate the associations between time-use compositions and health outcomes while controlling for covariates. The regression coefficient of the first *ilr* coordinate (e.g., sleep vs. the geometric mean of SB + LPA + MVPA) was interpreted to explore whether time spent in a particular behaviour was significantly associated with health outcomes. This meant four models were constructed for each outcome (i.e., a separate model with each behaviour represented by *ilr1*). The direction (positive or negative) and significance of the regression coefficient for *ilr1* can be used to infer whether changes in that behaviour are associated with an outcome when proportionally increasing or decreasing the other behaviours, however effect sizes are difficult to interpret [32]. To address this, models were then used to perform compositional isotemporal substitutions for each outcome [37]. To do this, the model predicted outcome for the mean time-use composition was first used as the reference composition. Subsequently, the model was used to make predictions for the changes in outcomes where time had been reallocated from one behaviour to another while time spent in the remaining behaviours were unchanged (e.g., 15 min/day reallocated from SB to MVPA while sleep and LPA remain unchanged). Estimates were made for reallocations in 5-min increments up to 30 min/day with Wald confidence intervals and were plotted to aid interpretation. Wald chi-square type II tests for fixed effects were performed using the car::Anova() function to test whether there was a significant association between the composition as a whole and each outcome. Note: results for the overall composition and isotemporal substitutions are invariant to how the *ilrs* were constructed and therefore consistent across models.

Cross-sectional models had fixed effects for time-use composition (*ilrs*), SES category, sex and age, and random intercepts to account for the clustering of participants within schools. Longitudinal models had fixed effects for time-use composition (*ilrs*), SES category, sex, age, initial health outcome for the relevant time period, and an indicator variable for time period (year 1, holidays, year 2). The inclusion of an indicator variable in the model for the time period functions as a proxy for other unobserved influences that may differ during each time period, such as diet, pubertal development, which could independently affect the outcomes.

and included random intercepts to account for multiple measurements for each participant (i.e., across the three time periods) and clustering of participants within schools. Longitudinal models displayed heteroscedastic residual variance across the time periods (higher variance during the holiday period). To improve model fit, residuals were allowed to vary across time periods using the weights = varIdent(form = ~ 1|time)) function [35]. This model was compared to the null model (with assumed homogeneity of variance) via the likelihood ratio test and demonstrated significantly better fit (*p* < 0.0001 for all outcomes) and was therefore used in all subsequent analyses. Unstandardised beta coefficients were reported for independent variables in models. To give an indication of the effect size the partR2 package [38] was used to estimate the marginal R [2] explained by the time-use composition in cross-sectional models. In longitudinal models the additional variance explained by the inclusion of time-use composition as a predictor was compared to the null model (without time-use composition).

## Results

Baseline participant characteristics are shown in Table 1. A total of 381 children enrolled for the study, with 23 dropping out before the study began. Between 259 and 281 (*n* = 259 for VO_2_max; *n* = 280 for %BF; *n* = 281 for zBMI) children provided complete data for demographic, time-use and outcome variables at baseline and were included in the cross-sectional models. Those excluded from analysis were more likely to be from the low-SES category (*p* < 0.01). No differences were observed between those included and those excluded from analysis for age or sex. Between 285 and 304 (*n* = 285 for VO_2_max; *n* = 303 for %BF; *n* = 304 for zBMI) participants provided data for demographic, time-use and outcome variables during at least one measurement period (year 1, holidays, year 2) and were included in the longitudinal models. Note that the number of participants was higher for the longitudinal models than for the cross-sectional model because participants only needed to contribute complete data for one measurement period to be included.

**Table 1:** Baseline participants characteristics

Participants (*n*)	281
Sex (n = M (%))	116 (41.3)
IRSD category (n (%))	
Low	93 (33.1)
Mid	97 (34.5)
High	91 (32.4)
Age (years, mean (SD))	9.38 (0.30)
zBMI (mean (SD))	0.48 (1.20)
%BF (mean (SD))	22.73 (8.83)
VO_2_max (mean (SD))	44.00 (3.40)
Sleep (mean (SD))	575 (43)
SB (mean (SD))	496 (87)
LPA (mean (SD))	290 (58)
MVPA (mean (SD))	79 (33)

Abbreviations: M = male; IRSD = Index of relative socio-economic disadvantage; zBMI = body mass index z-score; %BF = percent body fat; VO_2_max = maximal oxygen consumption. Note: arithmetic means and standard deviations shown for activity variables. Compositional mean: Sleep = 586, SED = 497, LPA = 286, MVPA = 72

### Cross-sectional associations between time-use compositions and health (baseline)

The time-use composition (ilrs) was statistically significantly associated with all outcomes (zBMI: χ²(3) = 13.64, p = < 0.001; %BF: χ²(3) = 31.4, p = < 0.001; VO_2_max: χ²(3) = 34.71, p = < 0.001; Table 2)). Results from the compositional mixed effect models with each behaviour as the pivot coordinate (Table 3) showed that, relative to the remaining behaviours, LPA was unfavourably associated with all health outcomes (zBMI: β = 1.22, *p* = 0.003; %BF: β = 9.27, *p* = 0.001; VO_2_max: β = -3.17, *p* = 0.004); MVPA was favourably associated with all health outcomes (zBMI: β = -0.62, *p* = 0.003); %BF: β = -5.81, *p* < 0.001; VO_2_max: β = 3.04, *p* < 0.001); Sleep was favourably associated with %BF (β = -10.4, *p* = 0.02);SB was unfavourably associated with %BF (β = 6.95, *p* = 0.02). However, sleep and SB displayed no significant associations with either zBMI or VO_2_max. The marginal R [2] attributable to time-use composition was higher for %BF and VO_2_max (9%, 10%, respectively) than for zBMI (4%).

**Table 2:** ANOVA tables of fixed effects for cross-sectional models

		zBMI		%BF		VO_2_max	
	df	χ²	P value	χ²	P value	χ²	P value
ilrs	3	13.64	**< 0.001**	31.4	**< 0.001**	34.71	**< 0.001**
SES category	2	6.89	**0.03**	17.76	**< 0.001**	7.98	**0.02**
Sex	1	2.81	0.09	1.63	0.20	10.27	**< 0.01**
Age	1	5.27	**0.02**	3.87	**0.049**	5.88	**0.02**

Type II Wald test. Abbreviations: ilrs = isometric log ratios; SES = socio-economic status; zBMI = body mass index z-score; %BF = percent body fat; VO2max = maximal oxygen consumption. Note: *p* < 0.05 shown in boldface

Note: results are equivalent regardless of which set of ilrs were used

**Table 3:** Cross-sectional associations between time-use compositions and health at T1

	zBMI			%BF			VO_2_max		
	Beta	Std. err	p value	Beta	Std. err	p value	Beta	Std. err	p value
ilr1 sleep	-0.75	0.66	0.26	-10.4	4.61	**0.02**	0.84	1.81	0.64
ilr1 SB	0.15	0.40	0.71	6.95	2.84	**0.02**	-0.72	1.11	0.52
ilr1 LPA	1.22	0.41	**< 0.01**	9.27	2.84	**< 0.01**	-3.17	1.10	**< 0.01**
ilr1 MVPA	-0.62	0.21	**< 0.01**	-5.81	1.46	**< 0.001**	3.04	0.56	**< 0.001**
SES mid	-0.08	0.19	0.67	-1.0	1.22	0.42	0.90	0.59	0.13
SES high	-0.52	0.21	**0.02**	-5.01	1.26	**< 0.01**	1.92	0.68	**< 0.01**
Sex-M	0.26	0.15	0.09	-1.39	1.09	0.20	1.29	0.40	**< 0.01**
Age	-0.54	0.24	**0.02**	-3.28	1.67	0.05	-1.47	0.61	**0.016**

Abbreviations: SB = sedentary behaviour; LPA = light physical activity; MVPA = moderate-to-vigorous physical activity; SES = socio-economic status; M = male; ilr = isometric-log ratio; zBMI = body mass index z-score; %BF = percent body fat; VO_2_max = maximal oxygen consumption. Note: *p* < 0.05 shown in boldface. Low SES and female were used as the referent groups, respectively

Note: four separate models were created with each model including a complete set of ilr coordinates (i.e., ilr1, ilr2 and ilr3). Only the regression coefficient corresponding to the pivot coordinate for each behaviour (ilr1) is displayed in Table 2. Results for additional terms are equivalent across models

**Fig. 1:**
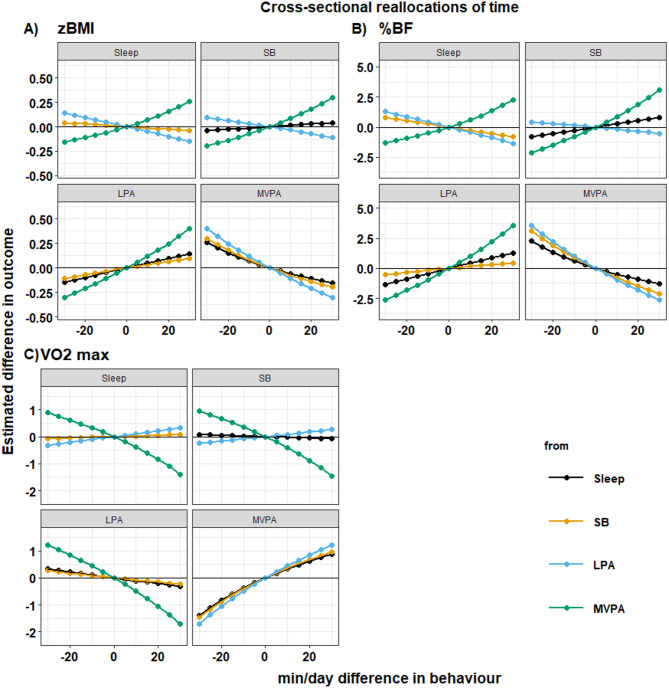
Estimated absolute difference in outcomes associated with pair-wise reallocations of time between behaviours using cross-sectional models. Subplots indicate reallocations for a single behaviour with increase (or decrease) at the expense of other behaviours. Abbreviations: SB, Sedentary behaviour; LPA, Light Physical Activity; MVPA, Moderate-to-Vigorous Physical Activity. Mean daily behaviour composition (min/day): Sleep = 586, SED = 497, LPA = 287, MVPA = 72. *n* = 281 (zBMI); *n* = 280 (%BF); *n* = 259 (VO_2_max)

Results from compositional isotemporal substitution models suggested that reallocating time toward MVPA from any behaviour was significantly and favourably associated with all three health outcomes. The strongest favourable associations were seen with reallocations from LPA to MVPA for all markers of health (Fig. 1 & Additional file 1). For example, 30 min/day more MVPA at the expense of LPA was associated with lower zBMI(-0.31 [95% CI: -0.47, -0.14]) and %BF (-2.65 [95% CI: -3.81, -1.49]), and higher VO2max (+ 1.23 ml/kg [95% CI: 0.78, 1.67]). Similarly, the strongest unfavourable associations were seen when reallocating time from MVPA to LPA for all health outcomes. However, the detrimental association of reallocations away from MVPA to other behaviours was larger than the favourable association observed when reallocating the equivalent amount of time towards MVPA in all instances. Conversely, benefits for health were smallest when reallocating time from sleep towards MVPA for all health outcomes, with associations for adiposity outcomes approximately half as strong when reallocating time from sleep toward MVPA when compared to LPA (30 min/day reallocation zBMI: -0.15 (95% CI: -0.31, -0.00); %BF: − 1.29 (95% CI: -2.36, 0.22)(Fig. 1 & Additional file 1). However, the differences for VO2max were less pronounced. Some beneficial associations were observed for reallocations not involving MVPA (for example, reallocating time from SB or LPA to sleep), however the magnitude of these associations was smaller than for any reallocations involving MVPA.

### Longitudinal associations between time-use compositions and health

Time-use composition was statistically significantly associated with rates of change in VO_2_max (χ²(3) = 26.72, p = < 0.01; Table 4), but not adiposity outcomes. However, time-use composition was approaching significance (*p* = 0.06) for %BF. Results from the compositional mixed-effects models with each behaviour as the pivot coordinate (Table 5) showed that MVPA (relative to the remaining behaviours) was significantly and favourably associated with rates of change for VO_2_max (β = 2.94, *p* < 0.001), with changes in %BF (β = -1.27, *p* = 0.06) also approaching statistical significance (Table 5). No other behaviours displayed significant associations with any outcome. Time-use composition explained 0.3% of the variance in BMI z-score, 2% in %BF, and 6% in VO₂max in longitudinal models, based on marginal R² values.

**Table 4:** ANOVA tables of fixed effects for longitudinal models

		zBMI		%BF		VO_2_max	
	df	χ²	P value	χ²	P value	χ²	P value
ilrs	3	1.89	0.60	7.34	0.06	26.72	**< 0.01**
SES category	2	7.47	**0.02**	2.23	0.33	2.43	0.30
Sex	1	0.46	0.50	1.07	0.30	1.99	0.16
Age	1	1.67	0.20	5.77	**0.02**	1.22	0.27
Starting outcome	1	26.16	**< 0.01**	40.64	**< 0.01**	59.97	**< 0.01**
Time	2	6.37	**0.04**	7.51	**0.02**	6.45	**0.04**

Type II Wald test. SES = socio-economic status; ilrs = isometric-log ratios; zBMI = body mass index z-score; %BF = percent body fat; VO_2_max = maximal oxygen consumption; df = degrees of freedom. Note: *p* < 0.05 shown in boldface; Year 1, low SES and female were used as the referent groups, respectively

**Table 5:** Longitudinal associations between time-use compositions and observed rates of change for health across three measurement periods

	zBMI			%BF			VO_2_max		
	Beta	St. err	p val	Beta	Std. err	p val	Beta	Std. err	p val
**ilr1 sleep**	0.08	0.24	0.74	-2.8	2.14	0.19	-2.72	2.14	0.21
**ilr1 SB**	0.05	0.15	0.76	1.82	1.37	0.19	-0.05	1.39	0.97
**ilr1 LPA**	-0.09	0.14	0.54	2.25	1.29	0.08	-0.17	1.22	0.89
**ilr1 MVPA**	-0.04	0.07	0.59	-1.27	0.66	0.06	2.94	0.67	**< 0.001**
**Time - HOL**	0.04	0.10	0.73	0.85	0.88	0.33	-1.08	0.91	0.24
**Time – Y2**	-0.11	0.05	**0.02**	-0.98	0.42	**0.02**	-0.99	0.41	**0.02**
**SES – mid**	-0.14	0.06	**0.01**	-0.69	0.49	0.16	0.33	0.57	0.56
**SES – high**	-0.14	0.06	**0.03**	-0.61	0.54	0.26	0.97	0.63	0.13
**Sex – M**	-0.03	0.05	0.50	0.46	0.45	0.3	0.61	0.43	0.16
**Age**	-0.10	0.07	0.20	-1.64	0.68	**0.02**	-0.71	0.64	0.27
**baseline measure**	-0.10	0.02	**< 0.001**	-0.17	0.03	**< 0.001**	-0.47	0.06	**< 0.001**

Abbreviations: SB = sedentary behaviour; LPA = light physical activity; MVPA = moderate-to-vigorous physical activity; SES = socio-economic status; M = male; ilr = isometric-log ratio; zBMI = body mass index z-score; %BF = percent body fat; VO_2_max = maximal oxygen consumption; Y2 = year 2, HOL = holidays. Note: *p* < 0.05 shown in boldface; Year 1, low SES and female were used as the referent groups, respectively

Note: four separate models were created with each model including a complete set of ilr coordinates (i.e., ilr1, ilr2 and ilr3). Only the regression coefficient corresponding to the pivot coordinate for each behaviour (ilr1) coordinate is displayed in Table 4. Results for additional terms are equivalent across models

**Fig. 2:**
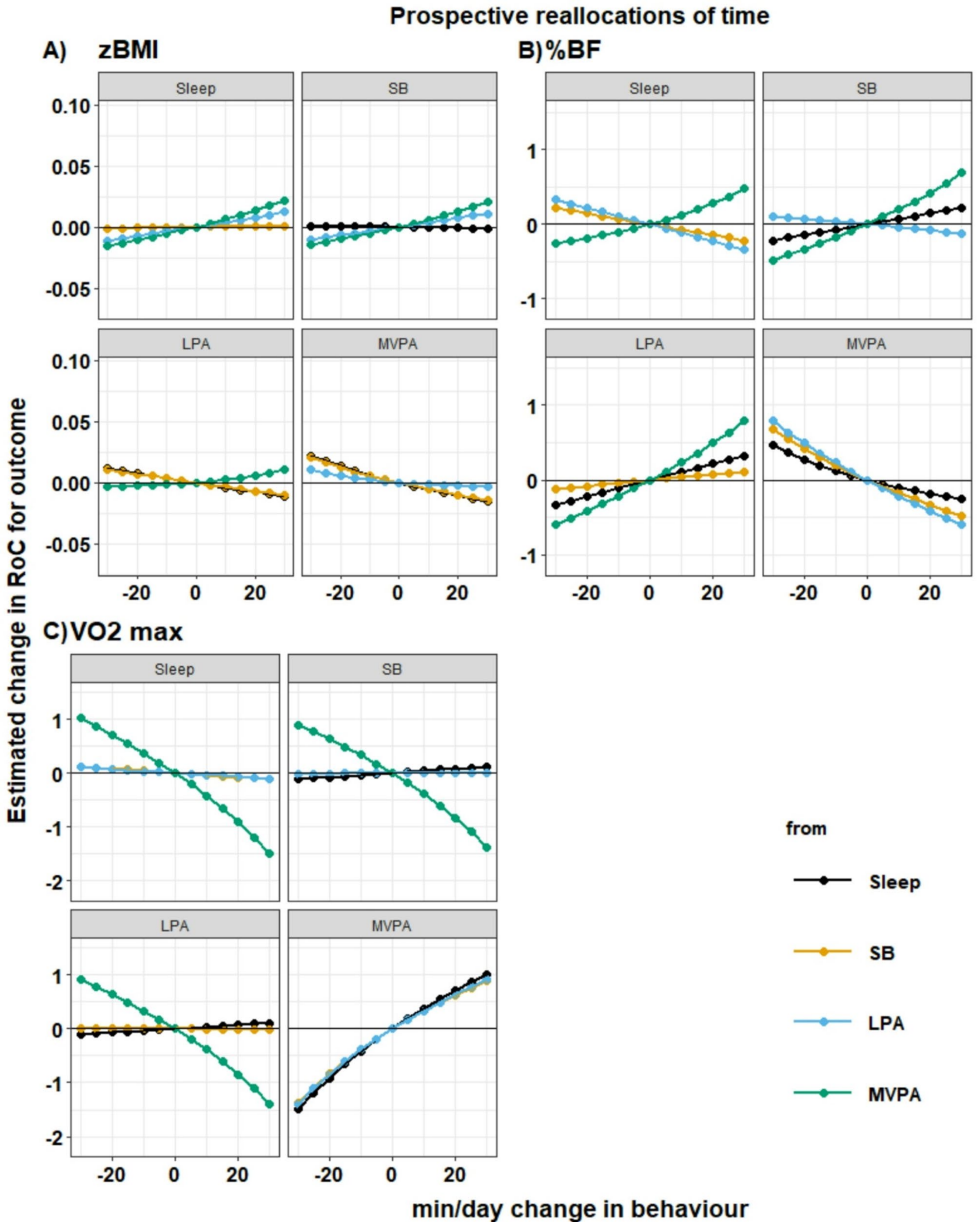
Estimated change in rates of change for outcomes associated with pair-wise reallocations of time between behaviours using longitudinal models. Subplots indicate reallocations for a single behaviour with increase (or decrease) at the expense of other behaviours. Mean daily behaviour composition (min/day): Sleep = 585, SED = 500, LPA = 283, MVPA = 72. Abbreviations: SB, Sedentary behaviour; LPA, Light Physical Activity; MVPA, Moderate-to-Vigorous Physical Activity. *n* = 305 (zBMI); *n* = 304 (%BF); *n* = 286 (VO_2_max)

Results from compositional isotemporal substitution models suggested that reallocating time toward MVPA from any behaviour was significantly and favourably associated with changes in RoC for VO_2_max (Fig. 2 & Additional file 1). Similar benefits were seen regardless of which behaviour was substituted. For example, a 30 min/day more MVPA was associated with RoC that were 1.01 (95% CI: 0.53, 1.50), 0.89 (95% CI: 0.51, 1.27), and 0.91 (95% CI: 0.38, 1.44) higher when time was substituted from sleep, SB and LPA, respectively. Reallocating time toward MVPA from SB and LPA, but not sleep, was significantly and favourably associated with changes in RoC for %BF. For example, 30 min/day more MVPA at the expense of SB was associated with a reduction in %BF RoC of -0.48 (95% CI: -0.86, -0.10) and 30 min/day more MVPA at the expense of LPA was associated with a reduction in RoC of -0.60 (95% CI: -1.14, -0.06). No other reallocations were significantly associated with changes in RoC for any health outcomes.

### Sensitivity analyses

In sensitivity analyses using a 5-part composition that considered MPA and VPA separately, cross-sectional models showed a statistically significant, favourable association between the pivot coordinate representing time in MPA with zBMI (β = -0.56, *p* = 0.03) and %BF (β = -3.79, *p* = 0.04), but not VO_2_max (Table S1, Additional file 2). The pivot coordinate representing time in VPA was statistically significantly associated with %BF (β = -1.44, *p* = 0.02) and VO_2_max (β = 1.09, *p* < 0.001), but not zBMI (Table S1, Additional File 2). Time reallocations suggested similar strength of relationships when reallocating time towards either MPA or VPA from other behaviours for zBMI or %BF. However, when considering VO_2_max, only reallocations towards VPA and not MPA from other behaviours were significantly associated with health (Additional file 1 & Figure S1, Additional File 2).

In longitudinal models, the pivot coordinate representing time in VPA was significantly, and favourably, associated with rates of change for both VO_2_max (β = 0.71, *p* < 0.01) and %BF (β = -0.58, *p* = 0.02) (Table S3, Additional File 2). When considering MPA separately to VPA, the pivot coordinate no longer displayed significant associations with any outcome; however, it did display favourable associations with VO_2_max that were bordering on statistical significance (β = 1.44, *p* = 0.06). Results from compositional isotemporal substitution models suggested that more time in either VPA (from sleep, SB or LPA) or MPA (from sleep or SB) was significantly and favourably associated with changes in RoC for VO_2_max (Additional file 1 & Figure S2, Additional File 2), however the largest changes were evident when increasing time in VPA. For example, 10 min/day more VPA at the expense of SB was associated with favourable changes to VO_2_max (increased RoC 0.53, 95% CI: 0.18, 0.88)) that were comparable to those associated with 30 min/day more MPA at the expense of SB (increased RoC 0.53, 95% CI: 0.00, 1.06) (Additional file 1 & Figure S2, Additional File 2). Only reallocations toward VPA (from either SB or LPA), and not MPA were significantly associated with favourable changes to %BF RoC. As with 4-part compositions, no time reallocations were significantly associated with changes to zBMI RoC.

### Discussion

This study applied CoDA to cross-sectional and longitudinal data to investigate how time-use compositions relate to CRF and adiposity in school-aged children. Cross-sectional results align with previous research suggesting that MVPA has the strongest favourable associations with health outcomes [9]. In addition to the favourable associations with MVPA, sleep was favourably associated with the health outcomes considered, whereas SB and LPA showed detrimental associations in some cross-sectional models. The detrimental associations between participation in SB or LPA and health in cross-sectional models aligns with previous research in this age group [9, 10]. While it remains unclear exactly why LPA is unfavourably associated with health in CoDA studies with children, it could simply be that this is because increased participation in LPA may take time away from other beneficial behaviours such as sleep or MVPA. However, in longitudinal models that used empirical changes in outcomes (as opposed to hypothetical changes in cross-sectional models), only time spent in MVPA - and in particular VPA - was significantly associated with changes in health. This suggests that the driver of changes to health may primarily be due to participation in higher intensity physical activities.

There remains a paucity of longitudinal studies that have used CoDA. However, our study supports some previous research that found cross-sectional relationships between lower-intensity activities and health being attenuated in longitudinal analyses when using CoDA [39–43]. The lack of significant associations for lower-intensity activities in the longitudinal models suggests that these behaviours may not provide sufficient stimulus to elicit meaningful changes in health outcomes over time. Conversely, MVPA involves higher energy expenditure and greater cardiorespiratory demand, and therefore may evoke physiological adaptations that results in improved CRF and adiposity outcomes.

Interestingly, this study found that associations between time-use composition and health were generally stronger for CRF than for adiposity outcomes. This may be due to a number of factors. Firstly, CRF may respond more quickly to changes in levels of MVPA than adiposity. A previous meta-analysis found that exercise interventions as short as 2–4 weeks in duration are effective in improving CRF [44]. Secondly, CRF may have a more direct causal relationship with higher intensity activity than adiposity does [45]which is likely impacted more strongly by other factors such as diet. It is important to note that physical activity only contributes a relatively small proportion to total energy expenditure [46]. Likewise, energy intake due to dietary factors also plays an important role in energy balance, and therefore markers of adiposity. Notably, in longitudinal models, reallocations toward MVPA resulted in relatively consistent improvements in VO_2_max, regardless of which behaviour was substituted, highlighting the central role of MVPA in improving CRF. Conversely, when considering %BF, reallocations were only significant when MVPA replaced SB or LPA, and not sleep. This suggests that increasing MVPA, while maintaining adequate sleep duration in children may be an important consideration in future health promotion efforts. Interestingly, no behaviours in longitudinal models were significantly associated with changes in zBMI within this cohort. Recent studies have noted a disagreement between zBMI and more direct estimates of fatness (such as %BF) in children of this age [47, 48]. Unlike %BF, zBMI does not provide any distinction between fat mass and other body tissue such as bone and muscle. It is also possible that zBMI is more strongly influenced by other factors, such as variations in child growth rates, in children of this age. Taken together, this could suggest that %BF is more sensitive to changes in time use than zBMI. The clinical significance of these findings is unclear, as there is no consensus on the minimal clinically important difference (MCID) for different markers of adiposity or CRF in this population [49]. In adults with overweight or obesity, it has been suggested that a reduction in %BF of 2% would be clinically important [50]. While a MCID of between + 1-3.5 ml/kg/min in VO_2_max has been suggested for adults with various clinical conditions [51–54]. However, it has also been noted that prevention of weight gain in the first instance is itself an important outcome [55]. This is particularly relevant to the current study, as this is where the largest associations were seen. It is also worth noting that many of the studies reporting MCID for adiposity are based on pharmaceutical studies that are accompanied by various side-effects, whereas participation in physical activity is often associated with additional benefits to other markers of health such as cardio-metabolic biomarkers, mental health and cognition [9, 10].

When considering VPA independently to MPA, our study found that VPA had particularly favourable associations with the health outcomes considered. This supports previous research that has shown VPA to have stronger relationships with health in children than MPA in studies using CoDA [9] and non-CoDA [56] approaches. In fact, in longitudinal models, the pivot coordinate representing MPA was not significantly associated with any health outcome. Despite this, isotemporal substitution models suggested that favourable changes to VO_2_max could still be seen when reallocating time from either sleep or SB to MPA. These seemingly contradictory results are due to the way changes to the pivot coordinate must be interpreted. The regression coefficient for the *ilr1* must be interpreted as changes in one behaviour relative to the geometric mean of the other behaviours, meaning that increases in the pivot coordinate representing MPA would be accompanied by proportional decreases in all behaviours, including VPA. Given VPA’s strong favourable association with VO_2_max, even small decreases would likely be detrimental and inhibit some of the benefit conferred through increases to MPA. However, the finding that CRF may still be improved through the accumulation of more MPA (when decreasing either sleep of SB) is important. While the benefits were lower on a minute-for-minute basis than those seen with VPA, MPA may be better tolerated by some children. This could make MPA a more feasible behaviour to target in health promotion efforts, particularly for those children unwilling to engage in more vigorous activity. Alternatively, given the strong relationship between VPA and both CRF and adiposity, it is possible that health practitioners should focus their attention on increasing participation in VPA, where even small increases are likely to be associated with improvements to health. The asymmetry in results for reallocations away from MVPA and VPA suggest that efforts should also be focused on preserving activity levels in children to mitigate detrimental changes to health that may be associated with noted decreases in MVPA due to temporal events (such as during weekends [30] and school holidays [57]), and more persistent changes that accompany aging into adolescence [58, 59].

### Strengths and limitations

A key strength of this study is the use of CoDA with a longitudinal study design. The study employed three distinct measurement periods for each participant in longitudinal models, which - in conjunction with the CoDA and multilevel modelling approach used - provides a robust understanding on the relationship between time-use compositions and changes in health outcomes. Whilst studies employing a CoDA approach have increased at a rapid rate in recent years, most of these are still limited by their cross-sectional nature [9]. Other strengths of this study include the objective measures of time-use behaviours, and valid and reliable measures of both adiposity and CRF.

However, limitations also exist. Like many studies, the COVID-19 pandemic impacted data collection and likely caused some data loss and increased participant drop-out rates. However, it must be noted that South Australia, where the study took place, was not as impacted by COVID-19 and associated public health restrictions as many parts of the world. Participants who did not provide complete data were also more likely to be from low-SES areas. This may limit the generalisability of findings. Our approach of creating composite time-use compositions for year 1 and year 2 also ignores any within-year variability for participants or specific behaviour patterns at the start or end of school year. There also exist no measures of time-use during winter where seasonal variations might exist. It is also possible that other factors that were not accounted for, such as dietary intake and puberty, are likely to explain some of the changes to health during the study period, particularly for adiposity outcomes. In our sample the mean composition (used as the reference) had 9.8 h/day of sleep, which is in the middle of the recommended range for Australian children of this age [60]. It is possible that relationship between sleep and health may differ for children who are not achieving the sleep guidelines.

## Conclusion

The results of the present study showed that many of the significant cross-sectional relationships between time-use composition and health in children were attenuated in longitudinal models. These findings emphasise the importance of prioritising MVPA, and especially VPA, in health promotion strategies for school-aged children. Notably, the lack of significant associations for LPA and SB in longitudinal models suggests that simply reducing sedentary time or increasing light activity may not be sufficient for meaningful health improvements. Therefore, future efforts should focus on encouraging MVPA with school-aged children to maximise benefits. More studies employing CoDA in longitudinal and randomised controlled study designs are needed to further explore causal relationships and refine recommendations for time-use reallocation in children’s daily routines.

## Electronic supplementary material

Below is the link to the electronic supplementary material.

Supplementary Material 1

Supplementary Material 2

Supplementary Material 3

## Data Availability

No datasets were generated or analysed during the current study.

## Abbreviations

%BF: percent body fat
20mSRT: 20-m shuttle run test
BMI: body mass index
CoDA: compositional data analysis
CRF: cardio-respiratory fitness
IRSD: Index of Relative Socio-economic Disadvantage
ilr: isometric log-ratio
LoH: Life on Holidays
LPA: light physical activity
MCID: minimal clinically important difference
MPA: moderate physical activity
MVPA: moderate- to vigorous- physical activity
RoC: rates of change
SB: sedentary behaviour
SES: socio-economic status
VO_2_max: volume of maximal oxygen uptake
VPA: vigorous physical activity
zBMI: BMI z-score
