# Supplementary material for: Cross-sectional and longitudinal associations between sleep, sedentary behaviour and physical activity with adiposity and cardio-respiratory fitness in school-aged children: a compositional data analysis
Source: J Act Sedentary Sleep Behav. 2025 Jul 11;4:11. doi: 10.1186/s44167-025-00082-y (PMC12247391; doi:10.1186/s44167-025-00082-y)
Supplement: Supplementary file 1 — Supplementary Material 1 [file 44167_2025_82_MOESM1_ESM.docx]

# Additional file 2.

## Model summaries

Model summary for cross-sectional and prospective models using 5-part compositions consisting of sleep, SB, LPA, MPA, VPA.

### Cross-sectional results

Table S1. Cross-sectional associations between time-use compositions and health at T1

|  | zBMI | | | %BF | | | VO2 max | | |  |
| --- | --- | --- | --- | --- | --- | --- | --- | --- | --- | --- |
|  | Beta | Std. err | p value | Beta | Std. err | p value | Beta | Std. err | p value |  |
| ilr1 sleep | -0.68 | 0.64 | 0.29 | -9.43 | 4.45 | **0.03** | 0.49 | 1.74 | 0.78 |  |
| ilr1 SB | 0.1 | 0.39 | 0.8 | 6.33 | 2.74 | **0.02** | -0.65 | 1.06 | 0.54 |  |
| ilr1 LPA | 1.25 | 0.43 | **<0.01** | 8.35 | 2.95 | **<0.01** | -1.82 | 1.15 | 0.11 |  |
| ilr1 MPA | -0.56 | 0.26 | **0.03** | -3.79 | 1.79 | **0.04** | 0.89 | 0.69 | 0.2 |  |
| ilr1 VPA | | -0.11 | 0.09 | 0.23 | -1.44 | 0.64 | **0.02** | 1.09 | 0.25 | **<0.001** |
| SES mid | -0.09 | 0.19 | 0.66 | -0.93 | 1.22 | 0.45 | 0.86 | 0.58 | 0.14 |  |
| SES high | -0.51 | 0.21 | **0.03** | -5.03 | 1.23 | **<0.01** | 2 | 0.67 | **<0.01** |  |
| Sex-M | 0.3 | 0.16 | 0.06 | -1.22 | 1.09 | 0.26 | 1.29 | 0.4 | **<0.01** |  |
| Age | -0.53 | 0.24 | **0.03** | -2.98 | 1.68 | 0.08 | -1.71 | 0.61 | **<0.01** |  |

Abbreviations: SB = sedentary behaviour; LPA = light physical activity; MPA = moderate physical activity; VPA = vigorous physical activity; SES = socio-economic status; ilr = isometric-log ratio; zBMI = body mass index z-score; %BF = percent body fat; VO2 max = maximal oxygen consumption. Note: p <0.05 shown in boldface; low SES and female were used as the referent groups, respectively.

Table S2. Anova tables of fixed effects for cross-sectional models

|  |  | zBMI | | %BF | | VO2 max | |
| --- | --- | --- | --- | --- | --- | --- | --- |
|  | df | χ² | P value | χ² | P value | χ² | P value |
| ilrs | 4 | 16.64 | **<0.01** | 34.63 | **<0.001** | 41.27 | **<0.001** |
| IRSD category | 2 | 6.52 | **0.04** | 18.99 | **<0.001** | 8.97 | **0.01** |
| Sex | 1 | 3.64 | 0.06 | 1.25 | 0.26 | 10.44 | **<0.01** |
| Age | 1 | 4.89 | **0.03** | 3.14 | 0.08 | 7.93 | **<0.01** |

Type II Wald test. Abbreviations: ilrs = isometric log ratios; SES = socio-economic status; zBMI = body mass index z-score; %BF = percent body fat; VO2 max = maximal oxygen consumption. Note: p <0.05 shown in boldface

### Prospective results

Table S3. Prospective associations between time-use compositions and observed rates of change for health across three measurement periods.

|  | **zBMI** | | | **%BF** | | | **VO2 max** | | |
| --- | --- | --- | --- | --- | --- | --- | --- | --- | --- |
|  | Beta | St. err | p val | Beta | Std. err | p val | Beta | Std. err | p val |
| **ilr1 sleep** | 0.06 | 0.23 | 0.79 | -3.06 | 2.06 | 0.14 | -2.14 | 2.05 | 0.3 |
| **ilr1 SB** | 0.06 | 0.15 | 0.69 | 2.03 | 1.32 | 0.13 | -0.35 | 1.33 | 0.79 |
| **ilr1 LPA** | -0.12 | 0.15 | 0.43 | 1.53 | 1.34 | 0.26 | 0.34 | 1.27 | 0.79 |
| **ilr1 MPA** | 0.02 | 0.09 | 0.84 | 0.08 | 0.79 | 0.92 | 1.44 | 0.77 | 0.06 |
| **ilr1 VPA** | -0.02 | 0.03 | 0.43 | -0.58 | 0.24 | **0.02** | 0.71 | 0.24 | **<0.01** |
| **Time - HOL** | 0.03 | 0.1 | 0.75 | 0.7 | 0.89 | 0.44 | -0.79 | 0.93 | 0.39 |
| **Time - Y2** | -0.11 | 0.05 | **0.02** | -0.96 | 0.43 | **0.03** | -1.02 | 0.41 | **0.01** |
| **SES - mid** | -0.13 | 0.06 | **0.02** | -0.66 | 0.53 | 0.21 | 0.33 | 0.57 | 0.56 |
| **SES - high** | -0.13 | 0.06 | **0.03** | -0.62 | 0.59 | 0.29 | 0.94 | 0.63 | 0.13 |
| **Sex - M** | -0.04 | 0.05 | 0.44 | 0.38 | 0.46 | 0.41 | 0.66 | 0.44 | 0.13 |
| **Age** | -0.1 | 0.07 | 0.2 | -1.66 | 0.68 | **0.02** | -0.7 | 0.64 | 0.27 |
| **Starting outcome** | -0.1 | 0.02 | **<0.001** | -0.17 | 0.03 | **<0.001** | -0.47 | 0.06 | **<0.001** |

Abbreviations: SB = sedentary behaviour; LPA = light physical activity; MPA = moderate physical activity; VPA = vigorous physical activity; SES = socio-economic status; ilr = isometric-log ratio; zBMI = body mass index z-score; %BF = percent body fat; VO2 max = maximal oxygen consumption; Y2 = year 2, HOL = holidays. Note: p < 0.05 shown in boldface; Year 1, low SES and female were used as the referent groups, respectively.

Table S4.

|  |  | zBMI | | %BF | | VO2 max | |
| --- | --- | --- | --- | --- | --- | --- | --- |
|  | df | χ² | P value | χ² | P value | χ² | P value |
| ilrs | 4 | 2.25 | 0.69 | 10.71 | **0.03** | 26.57 | **<0.001** |
| SES category | 2 | 7.06 | **0.03** | 1.87 | 0.39 | 2.31 | 0.31 |
| Sex | 1 | 0.59 | 0.44 | 0.67 | 0.41 | 2.26 | 0.13 |
| Age | 1 | 1.67 | 0.2 | 5.88 | **0.02** | 1.2 | 0.27 |
| Starting outcome | 1 | 25.63 | **<0.001** | 41.35 | **<0.001** | 59.39 | **<0.001** |
| Time | 2 | 6.18 | **0.046** | 6.63 | **0.04** | 6.32 | **0.04** |

Type II Wald test. Abbreviations: ilrs = isometric-log ratios; SES = socio-economic status; zBMI = body mass index z-score; %BF = percent body fat; VO2 max = maximal oxygen consumption; df = degrees of freedom. Note: p < 0.05 shown in boldface; Year 1, low SES and female were used as the referent groups, respectively.

## Reallocation figures


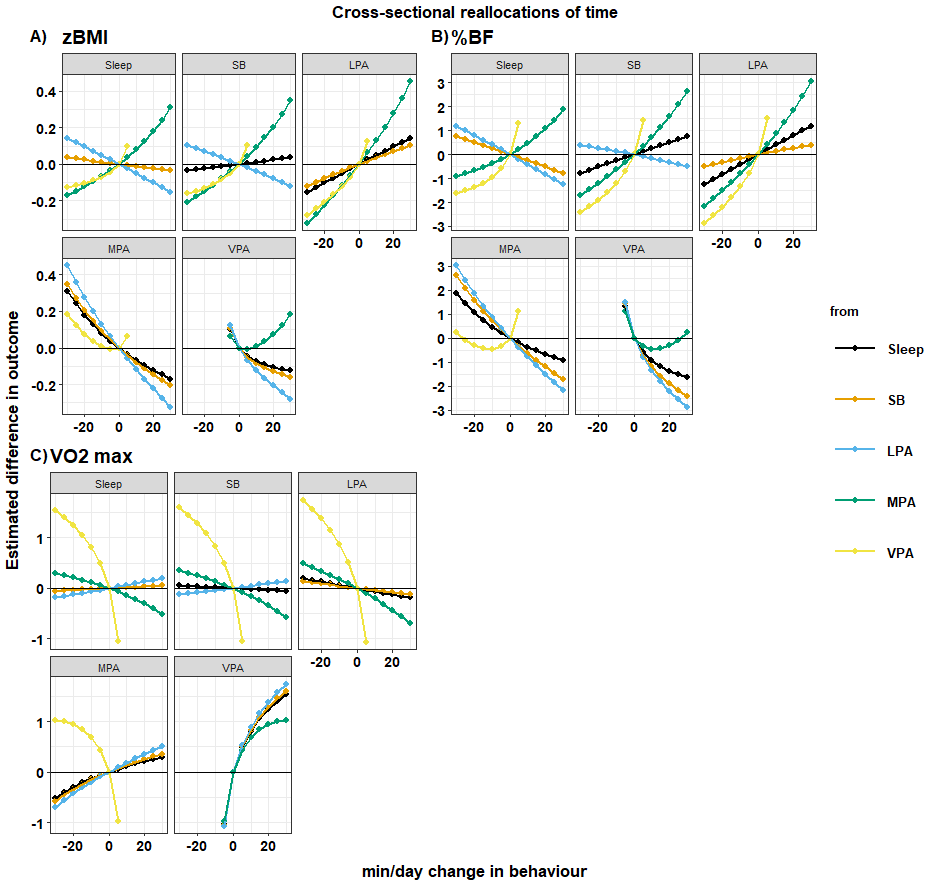

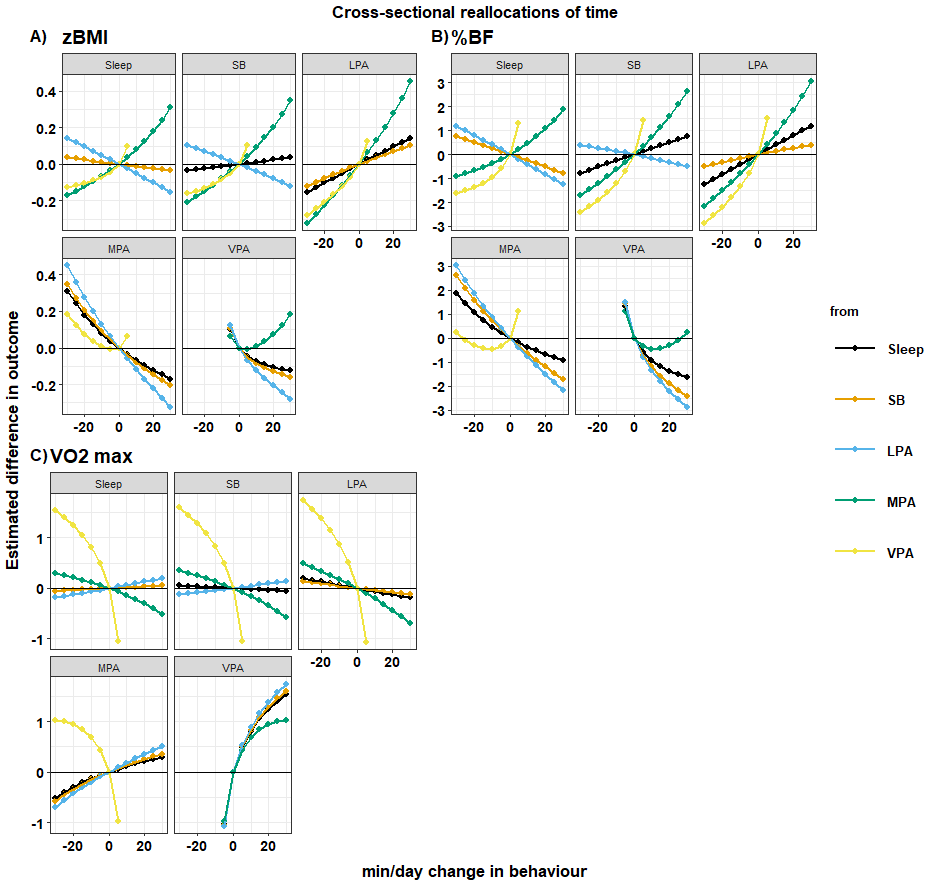


Figure S1. Estimated absolute difference in outcomes associated with pair-wise reallocations of time between behaviours using cross-sectional models. Abbreviations: SB, Sedentary behaviour; LPA, Light Physical Activity; MVPA, Moderate-to-Vigorous Physical Activity. Mean daily behaviour composition (min/day): Sleep = 587, SED = 498, LPA = 286, MPA = 61, VPA = 8


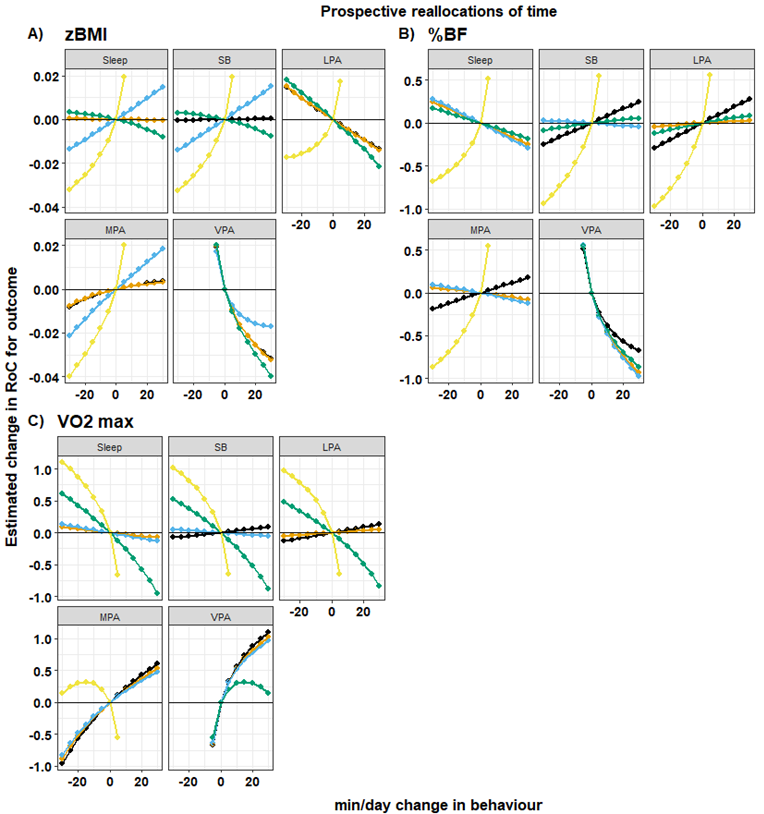

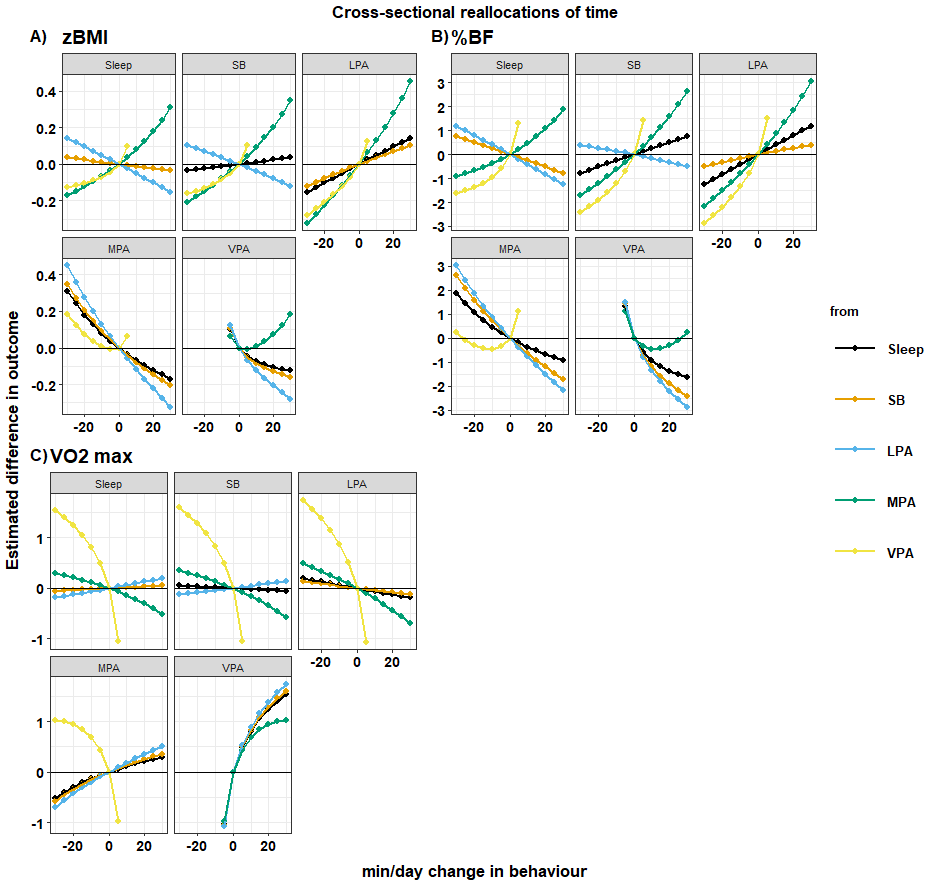


Figure S2. Estimated change in rates of change for outcomes associated with pair-wise reallocations of time between behaviours using cross-sectional models. Abbreviations: SB, Sedentary behaviour; LPA, Light Physical Activity; MVPA, Moderate-to-Vigorous Physical Activity. Mean daily behaviour composition (min/day): Sleep = 586, SED = 502, LPA = 283, MPA = 61, VPA = 8
